# Prognostic significance of CD8+ T-cells density in stage III colorectal cancer depends on SDF-1 expression

**DOI:** 10.1038/s41598-020-80382-2

**Published:** 2021-01-12

**Authors:** Alexandros Lalos, Ali Tülek, Nadia Tosti, Robert Mechera, Alexander Wilhelm, Savas Soysal, Silvio Daester, Venkatesh Kancherla, Benjamin Weixler, Giulio C. Spagnoli, Serenella Eppenberger-Castori, Luigi Terracciano, Salvatore Piscuoglio, Markus von Flüe, Alberto Posabella, Raoul A. Droeser

**Affiliations:** 1 University Center for Gastrointestinal and Liver Diseases, Clarunis, University of Basel, Basel, Switzerland; 2 Institute of Pathology and Medical Genetics, University Hospital Basel, Basel, Switzerland; 3 Department of Colorectal Surgery, Royal Prince Alfred Hospital, Sydney, Australia; 4 Department of Surgery, Charité University Hospital, Campus Benjamin Franklin, Berlin, Germany; 5 Department of Biomedicine, University Hospital Basel, Basel, Switzerland; 6 Visceral Surgery Research Laboratory, Clarunis, Department of Biomedicine, University of Basel, Basel, Switzerland

**Keywords:** Biomarkers, Medical research, Oncology, Immunochemistry

## Abstract

Since colorectal cancer (CRC) remains one of the most common malignancies, a tremendous amount of studies keep taking place in this field. Over the past 25 years, a notable part of the scientific community has focused on the association between the immune system and colorectal cancer. A variety of studies have shown that high densities of infiltrating CD8+ T-cells are associated with improved disease-free and overall survival in CRC. Stromal cell-derived factor-1 (SDF-1) is a protein that regulates leukocyte trafficking and is variably expressed in several healthy and malignant tissues. There is strong evidence that SDF-1 has a negative prognostic impact on a variety of solid tumors. However, the existing data do not provide sufficient evidence that the expression of SDF-1 has an influence on CRC. Knowing nowadays, that the microenvironment plays a crucial role in the development of cancer, we hypothesized that the expression of SDF-1 in CRC could influence the prognostic significance of CD8+ T-cells, as an indicator of the essential role of the immune microenvironment in cancer development. Therefore, we explored the combined prognostic significance of CD8+ T-cell density and SDF-1 expression in a large CRC collective. We analyzed a tissue microarray of 613 patient specimens of primary CRCs by immunohistochemistry (IHC) for the CD8 + T-cells density and the expression of SDF-1 by tumor cells and tumor-infiltrating immune cells. Besides, we analyzed the expression of SDF-1 at the RNA level in The Cancer Genome Atlas cohort. We found that the combined high CD8+ T-cell infiltration and expression of SDF-1 shows a favorable 5-year overall survival rate (66%; 95% CI 48–79%) compared to tumors showing a high expression of CD8+ T-cell only (55%; 95% CI 45–64%; *p* = 0.0004). After stratifying the patients in nodal negative and positive groups, we found that the prognostic significance of CD8+ T-cell density in nodal positive colorectal cancer depends on SDF-1 expression. Univariate and multivariate Hazard Cox regression survival analysis considering the combination of both markers revealed that the combined high expression of SDF-1 and CD8+ T-cell density was an independent, favorable, prognostic marker for overall survival (HR = 0.34, 95% CI 0.17–0.66; *p* = 0.002 and HR = 0.45, 95% CI 0.23–0.89; *p* = 0.021, respectively). In our cohort there was a very weak correlation between SDF-1 and CD8+ T-cells (r_s_ = 0.13, *p* = 0.002) and in the trascriptomic expression of these two immune markers display a weak correlation (r_s_ = 0.28, *p* < 0.001) which was significantly more pronounced in stage III cancers (r_s_ = 0.40, *p* < 0.001). The combination of high CD8+ T-cell density and expression of SDF-1 represents an independent, favorable, prognostic condition in CRC, mostly in patients with stage III disease.

## Introduction

Colorectal cancer (CRC) is the third most common malignancy to be diagnosed. Furthermore, CRC remains the second most common cause of death from cancer worldwide, despite the screening and new methods of treatment^1,2^. These facts explain why so many studies have taken place to identify the mechanisms of CRC development. Understanding these features could lead to new concepts in the approach of diagnosis, prognosis, and even treatment of CRC.

As with the majority of cancers, the Tumor Node Metastasis (TNM) classification serves as the gold standard tool for the staging of CRC^3^. By every newly diagnosed CRC, an interdisciplinary team of specialists in several fields (Visceral Surgery, Oncology, Gastroenterology, Radiology/Nuclear Medicine, Radiation Oncology and Pathology) takes into account the TNM classification to determine the treatment. According to this system, we decide which patients are suitable for surgical resection, which are candidates for adjuvant chemotherapy following the resection of the primary tumor and which are palliative cases^4,5^. However, we repeatedly observe that patients with identical stages and treatments have a completely different outcome in terms of survival and recurrence. This fact indicates that TNM classification alone in the vast majority of cases is not sufficient for the prognosis of colorectal cancer^6,7^. Subsequently, an enormous amount of studies have investigated and still investigate other features, which could be an excellent additional tool for this classification. The crucial role of the microenvironment in CRC was thoroughly explored and showed that the high immune cell infiltration by cytotoxic CD8+ T-cells and memory CD45RO+ T-cells has a favorable prognostic significance^8–12^.

CD8 (cluster of differentiation 8) is a well-known protein that serves as a co-receptor for the T-cell receptor (TCR) and binds to the major histocompatibility complex (MHC) molecule^13^. When the cytotoxic T cells are combined with CD8 surface protein, produce the CD8+ T cells and play a vital role in antigen recognition. Naito et al.^14^ proved that the infiltration of tumors with CD8+ T-cells has a beneficial prognostic influence in CRC. Since then, a substantial number of studies have also examined the role of CD8+ T-cells, and nowadays, the scientific society has accepted its positive prognostic impact on CRC^15–19^. These results highlight the fact that the infiltration of the tumor with CD8+ T-cells is associated with a better prognosis not only in CRCs but also in other malignancies such as lung^20^, renal^21^ and endometrial^22^.

Following these positive and promising results, various immune markers were investigated with the aim of finding a better and more sufficient way to evaluate the prognosis of CRC. The stromal cell-derived factor 1 (SDF-1) is a chemokine protein, which is strongly chemotactic for lymphocytes^23^. SDF-1 and one of its receptors, CXCR4, have been shown to play a crucial role in the tumor-stromal communication affecting cancer growth, angiogenesis, and metastasis formation^24^. Samarenda et al. performed a meta-analysis of 38 studies that evaluated the association between SDF-1 expression and cancer survival. The authors showed that a high SDF-1 expression was associated with significantly reduced overall survival in patients with lung, pancreatic, and esophagus-gastric cancer. Yet, there was no correlation between SDF-1 expression and overall survival in colorectal cancer^25^.

Taking these facts into account, we conducted this study in order to evaluate the prognostic significance of these two markers alone and in combination. We hypothesized that the expression of SDF-1 could influence the prognostic significance of CD8+ T-cell density. The secondary aim of the study was to explore thoroughly the interaction between the different particles of the immune microenvironment in CRC^26–29^.

## Methods

### Tissue microarray construction

In our study, we included 613 patients with unselected, clinically annotated primary CRC specimens in a tissue microarray (TMA). Our study had the approval of the local ethics committee (Ethikkommission Nordwest- und Zentralschweiz). All methods were carried out in accordance with relevant guidelines and regulations. As far as it concerns the technique used for the TMA construction, we have described this in previous studies of our team^30^. The TMAs were constructed by the specialists of the Pathology Biobank at the University Hospital of Basel (Basel, Switzerland). Unselected, nonconsecutive, formalin-fixed, paraffin-embedded primary colorectal cancer tissue blocks were used as donor blocks. Tissue cylinders with a diameter of 1 mm were punched from morphologically representative areas of each donor block and brought into one recipient paraffin block (30 × 25 mm). Each punch was derived from the center of the tumor in an area with no necrosis so that each TMA spot consisted of more than 50% tumor cells.

### Clinico-pathological features

Clinico-pathological data were collected retrospectively in a non-stratified and random manner. Annotation included patient age and gender, tumor diameter in mm, site of the tumor, pT-stage, pN-stage, grade, stage according to TNM-classification, tumor border configuration (infiltrative vs pushing), vascular invasion, overall survival time (months), 5-years survival in % (95% CI) and the presence of peri-tumoral lymphocytic inflammation at the invasive tumor front (Table 1). Tumor border configuration and peri-tumoral lymphocytic inflammation were evaluated using the original hematoxylin–eosin slides of the resection specimens corresponding to each TMA punch.

**Table 1:** Characteristics of CRC patient cohort (n = 613).

Characteristics	N or mean	(% or range)
Age, years (median, mean)	70, 68.9	36–96
Tumor size in mm (median, mean)	50, 50.1	4–160
**Sex**		
Female	326	53.2
Male	287	46.8
**Anatomic site of the tumor**		
Left-sided	428	69.8
Right-sided	183	29.9
Missing values	2	0.3
**T stage**		
T1	31	5.1
T2	83	13.5
T3	406	66.2
T4	79	12.9
Missing values	14	2.3
**N stage**		
N0	317	51.7
N1	160	26.1
N2	119	19.4
Missing values	17	2.8
**Tumor grade**		
G1	14	2.3
G2	552	90.0
G3	33	5.4
Missing values	14	2.3
**UICC TNM classification**		
Stage I	84	13.7
Stage II	227	37.0
Stage III	279	45.5
Missing values	17	3.8
**Tumor border configuration**		
Infiltrative	417	68.0
Pushing	180	29.4
Missing values	16	2.6
**Vascular invasion**		
No	425	69.3
Yes	174	28.4
Missing values	14	2.3
**Microsatellite stability**		
Proficient	539	87.9
Deficient	74	12.1
Rectal cancers	246	40.1
Rectosigmoid cancers	44	7.2
Overall survival time (months)	58.4	0–152
5-years survival % (95%CI)	0.47	0.43—0.51
CXCR4 histoscore	156.5	0—300
pCXCR4 histoscore	32.2	0—300
CXCR4 TIC	74.2	0—1000
pCXCR4 TIC	4.8	0—79

Age and tumor size were evaluated using the Kruskal–Wallis test. Gender, anatomical site, T stage, N stage, grade, vascular invasion, and tumor border configuration were analyzed using the χ^2^ test. Survival analysis was performed using the Kaplan–Meier method.

### Immunohistochemistry

We used standard indirect immunoperoxidase procedures (IHC; ABC-Elite, Vector Laboratories, Burlingame, CA) as we have already described in previous studies of our team^31^. Our specialists dewaxed and rehydrated slides in distilled water. Afterward, endogenous peroxidase activity was blocked using 0.5% H_2_O_2_. Sections were incubated with 10% normal goat serum (DakoCytomation, Carpinteria, CA) for 20 min and incubated with primary antibody at room temperature. We used primary antibodies that were specific for CD8 (Ventana 790-4460) and SDF-1 (Abcam ab9797). Subsequently, these parts were incubated with peroxidase-labeled secondary antibody (DakoCytomation) for 30 min at room temperature. For visualization of the antigen, these parts were immersed in 3-amino-9-ethylcarbazole plus substrate-chromogen (DakoCytomation) for 30 min and counterstained with Gill’s hematoxylin.

### Evaluation of immunohistochemistry

Two trained research fellows [A.L. and A.T.] performed immunohistochemical analysis, and an experienced pathologist [L.T.] validated the date independently. Histoscores for expression by tumor cells were obtained by multiplying percentages of positive cells by staining intensities (0 = negative, 1 = weak, 2 = moderate, 3 = strong). Tumor-infiltrating immune cells (TICs) were counted for each punch (approximately one high power [20 ×] field).

### Public database analysis

Correlations using the RNA-sequencing from The Cancer Genome Atlas (TCGA) were performed. Briefly, colorectal cancer clinical information available for 597 patients was retrieved from the Human Protein Atlas database [https://www.proteinatlas.org/ENSG00000132688-NES/pathology/tissue/colorectal+cancer]. Gene Expression levels (FPKM values) for the genes were downloaded using TCGAbiolinks R package.

### Statistical analysis

We explored associations with survival using the Cox proportional hazard regression model. Cut-off values used to classify CRC with low or high immune cell infiltration were available from previous publications^32^ or generated by applying regression tree analysis. Threshold value for CD8+ was 10 cells/TMA-punch. Threshold value for SDF-1 histoscore was 200 (= 75th percentile). Chi-square, Fisher’s exact, and Kruskal–Wallis tests were used to determine the association of SDF-1 tumor expression and CD8+ T-cell infiltration and clinical-pathological features.

For survival analysis, the study population was randomly subset into a test and a validation group. Furthermore, in order to get more information we furtherly stratified the patients according to their tumor stage. The Kaplan–Meier survival curves were compared accordantly to the log-rank test. Further, analysis included all four combinations possible (SDF-1^high^/CD8 + ^high^, SDF-1^high^/CD8 + ^low^, SDF-1^low^/CD8 + ^high^, SDF-1^low^/CD8 + ^low^) and the resulting Kaplan–Meier curves were compared pairwise and the p-value was adjusted for multiple comparisons according to the Benjamini and Hochberg (1995).

The assumption of proportional hazards was verified for all markers by analyzing correlation of Schoenfeld residuals and ranks of individual failure times. Any missing clinicopathological information was assumed to be missing at random. Subsequently, SDF-1 expression and CD8 + cell density data were entered into multivariate Cox regression analysis and hazard ratios (HR) and 95% confidence intervals (CI) were used to determine prognostic effects on survival time. *P* values < 0.05 were considered statistically significant. Statistical analyses were made using STATA software version 13 (StataCorp, College Station, TX, USA) and with the Statistical Package Software R (version 4.0.2, http//.r-project.org).

### Ethics approval and consent to participate

Our study was reviewed and approved by Ethics Committee of Basel, EKBB, number EKBB 361/12. The patients/participants provided their written informed consent to participate in this study.

### Consent for publication

The consent was waived by the ethics committee (Ethikkommission Nordwest- und Zentralschweiz).

## Results

### Patient and tumor characteristics

In our study we included 613 patients with CRC with a median age of 70 years (range 36–96). 53.2% of the patients were female. In 70% of the patients CRC was located in the left hemicolon or rectum and in the remaining 30% in the right hemicolon. According to the TNM-classification, most of the tumors were of pT3/pT4 stage (n = 485), with the minority of cancers being pT1/pT2 (n = 114). Among the malignancies under evaluation, 317 were pN0, 160 pN1, and 119 pN2 cases. Tumor grade was G1 in 14 cases, G2 in 552 cases, and G3 in 33 cases. Furthermore 417 showed infiltrating tumor border configuration, while in the majority of cases (n = 425) vascular invasion was absent. Most of the tumors belonged to the microsatellite stable subgroup (n = 539). Within the follow-up period, median disease-specific survival was 58.4 months (range 0–152), while mean 5-year-survival was 47% (95% CI 43–51%). In Table 1, we summarized the clinico-pathological characteristics of these patients.

### Association of clinicopathological features with SDF-1 expression and CD8+ T cell density

**Figure 1:**
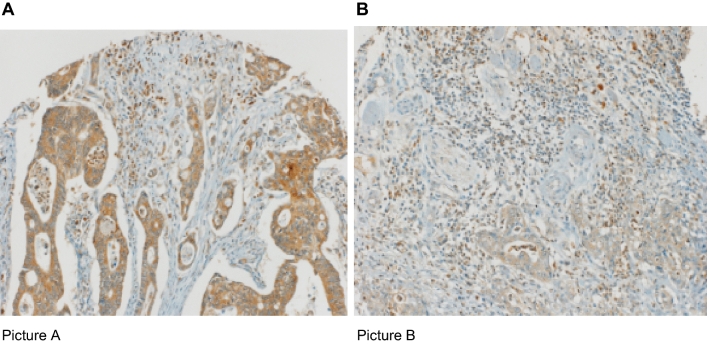
Examples of high SDF-1 expression (**A**) and low SDF-1 expression (**B**) in CRC biopsies with high CD8+ T-cell infiltration (× 200 magnification).

In Fig. 1, we illustrate 2 representative pictures of low and high expression of SDF-1 in biopsies with high CD8 + T-cell infiltration in CRC. Furthermore, we created a table, that demonstrates the clinicopathological features under investigation and their relation to the four subgroups identified by SDF-1 expression and CD8 + T-cell density (SDF-1^high^/CD8 + ^high^, SDF-1^high^/CD8 + ^low^, SDF-1^low^/CD8 + ^high^, SDF-1^low^/CD8 + ^low^) as absolute numbers and percentages (Table 2). As it is presented in our study, we observed that cases with SDF-1^high^/CD8 + ^high^ were characterized by a significantly lower N-stage (*p* < 0.001) in comparison to the cases with SDF-1^high^/CD8 + ^low^. In contrast, we demonstrated that T stage, tumor grade and vascular invasion did not significantly differ in CRC with different immune environment profiles.

**Table 2:** Association of SDF-1 + tumor expression and CD8+ low and high immune cell density with clinicopathological features in CRC (n = 613).

	SDF-1^high^/CD8 + ^high^		SDF-1^high^/CD8 + ^low^		SDF-1^low^/CD8 + ^high^		SDF-1^low^/CD8 + ^low^		*p* value
	N = 35	(100%)	N = 121	(100%)	N = 98	(100%)	N = 359	(100%)	
**Age**									
Years, mean ± SD	68.5	± 10.6	69.4	± 10.9	67.6	± 12.0	69.1	± 11.0	0.751
**Tumor diameter**									
mm, mean ± SD	53.2	± 14.2	48.2	± 16.1	52.5	24.4	49.9	± 20.2	0.253
**Gender**									
Female	25	71.4	60	49.6	52	53.1	189	52.6	
Male	10	28.6	61	50.4	46	46.9	170	47.4	0.146
**Tumor location**									
Left-sided	23	65.7	97	80.2	64	65.3	244	67.9	**0.046**
Right-sided	12	34.3	24	19.8	33	33.7	114	31.8	
Missing values	0	0.0	0	0.0	1	1.0	1	0.3	
**Histologic subtype**									
Mucinous	2	5.7	3	2.5	3	3.1	22	6.1	0.292
Non-mucinous	33	94.3	118	97.5	95	96.9	337	93.9	
**pT stage**									
pT1-2	9	25.7	23	19.0	23	23.5	59	16.4	0.177
pT3-4	24	68.6	96	79.3	70	71.4	295	82.2	
Missing values	2	5.7	2	1.7	5	5.1	5	1.4	
**pN stage**									
pN0	23	65.7	61	50.4	67	68.4	166	46.2	**< 0.001**
pN1-2	12	34.3	57	47.1	27	27.5	183	51.0	
Missing values	0	0.0	3	2.5	4	4.1	10	2.8	
**Tumor grade**									
G1	0	0.0	2	1.7	2	2.0	10	2.8	0.097
G2	32	91.4	112	92.5	79	80.6	329	91.6	
G3	1	2.9	5	4.1	12	12.3	15	4.2	
Missing values	2	5.7	2	1.7	5	5.1	5	1.4	
**Vascular invasion**									
Absent	26	74.3	88	72.7	67	68.4	244	68.0	0.559
Present	7	20.0	31	25.6	26	26.5	110	30.6	
Missing values	2	5.7	2	1.7	5	5.1	5	1.4	
**Tumor border**									
Pushing	13	37.1	37	30.6	39	39.8	91	25.4	**0.014**
Infiltrating	20	57.2	82	67.7	54	55.1	261	72.7	
Missing values	2	5.7	2	1.7	5	5.1	7	1.9	
**PTL inflammation**									
Absent	20	57.2	94	77.7	67	68.4	282	78.5	**0.051**
Present	13	37.1	25	20.6	26	26.5	72	20.1	
Missing values	2	5.7	2	1.7	5	5.1	5	1.4	
**Microsatellite stability**									
Deficient	3	8.6	5	4.1	18	18.4	48	13.4	**0.004**
Proficient	32	91.4	116	95.9	80	81.6	311	86.6	
**5-year survival rate**									
(95% CI)	0.66	(0.48–0.79)	0.40	(0.310.48)	0.55	(0.45–0.64)	0.45	(0.40–0.50)	**0.0004**
**CXCR4 histoscore**									
mean ± SD	203.5	± 100.2	177.3	± 95.5	152.9	± 94.4	143.6	± 99.7	**0.001**
**CXCR4 TIC**									
mean ± SD	50.7	± 62.3	53.5	± 130.7	121.9	± 208.8	70.7	± 145.8	**0.016**
**pCXCR4 histoscore**									
mean ± SD	76.3	± 97.3	32.4	± 64.2	54.1	± 81.8	20.9	± 49.2	**0.0002**
**pCXCR4 TIC**									
mean ± SD	6.2	± 9.1	4.3	± 10.4	7.6	± 14.0	4.0	± 6.8	**0.001**

Variables are indicated as absolute numbers, %, median or range; age and tumor size were evaluated using the Kruskal–Wallis test. Gender, anatomical site, T stage, N stage, grade, vascular invasion, and tumor border configuration were analyzed using the χ^2^ test. Survival analysis was performed using the Kaplan–Meier method. Significant *p *values are bold.

Furthermore, the nature of tumor border (pushing vs. infiltrating) has been reported to impact on CRC prognosis, with the infiltrating tumor border being associated with poor survival^32^. In our cohort, infiltrating tumor border was detected with significantly (*p* = 0.014) higher frequency in tumors with low density of the CD8 + T-cells. Interestingly, we detected a strong tendency towards statistical significance (*p* = 0.051) regarding the presence of PTL inflammation, which was higher in the group of SDF-1^high^/CD8 + ^high^ in contrast to SDF-1^high^/CD8 + ^low^. This fact highlights the importance of the microenvironment and supports the hypothesis of an effective antigen-specific immune response. However, this can only be considered as a trend with a *p* > 0.05.

### Spearman’s correlation analysis of SDF-1, CD8 + T-cell and markers of the microenvironment

In order to understand better the microenvironment in the context of SDF-1 expression and CD8+ T-cell density we performed a Spearman’s correlation analysis with a panel of immune, cell signaling markers or growth factors on protein (TMA data; Table 3) and considered correlations above 0.35 to be relatively strong, correlations between 0.15 and 0.35 to be moderate, and those below 0.15 to be weak. Finally, SDF-1 tumor expression moderately correlated with CXCR4 tumor expression. SDF-1 expression by tumor-infiltrating immune cells (TIC) showed a strong correlation with CXCR4 positive TIC and a moderate correlation with CD8 T-cell density as well as pCXCR4 positive TIC on a protein level (Table 3).

**Table 3:** Spearman’s correlation analysis of SDF-1 protein expression with CXCR4, pCXCR4 and CD8.

	SDF-1 + tumor expression	SDF-1 + TIC	CD8	CXCR4 tumor expression	pCXCR4 tumor expression	CXCR4 + TIC	pCXCR4 TIC
SDF-1 + tumor expression	1.000						
SDF-1 + TIC	− 0.1143 0.0149	1.0000					
CD8	− 0.0359 0.4455	0.1648 0.0004	1.0000				
CXCR4 tumor expression	0.1864 0.0001	0.0571 0.2252	0.1244 0.0080	1.0000			
pCXCR4 tumor expression	0.0296 0.5291	− 0.0245 0.6035	0.2601 0.0000	0.2764 0.0000	1.0000		
CXCR4 + TIC	− 0.0957 0.0418	0.3752 0.0000	0.2128 0.0000	0.1174 0.0124	0.1020 0.0300	1.0000	
pCXCR4 + TIC	− 0.0806 0.0865	0.1572 0.0008	0.2034 0.0000	0.1131 0.0160	0.2725 0.0000	0.3706 0.0000	1.0000

### Synergistic prognostic significance of CD8+ T-cell density and expression of SDF-1 in the CRC microenvironment

**Figure 2:**
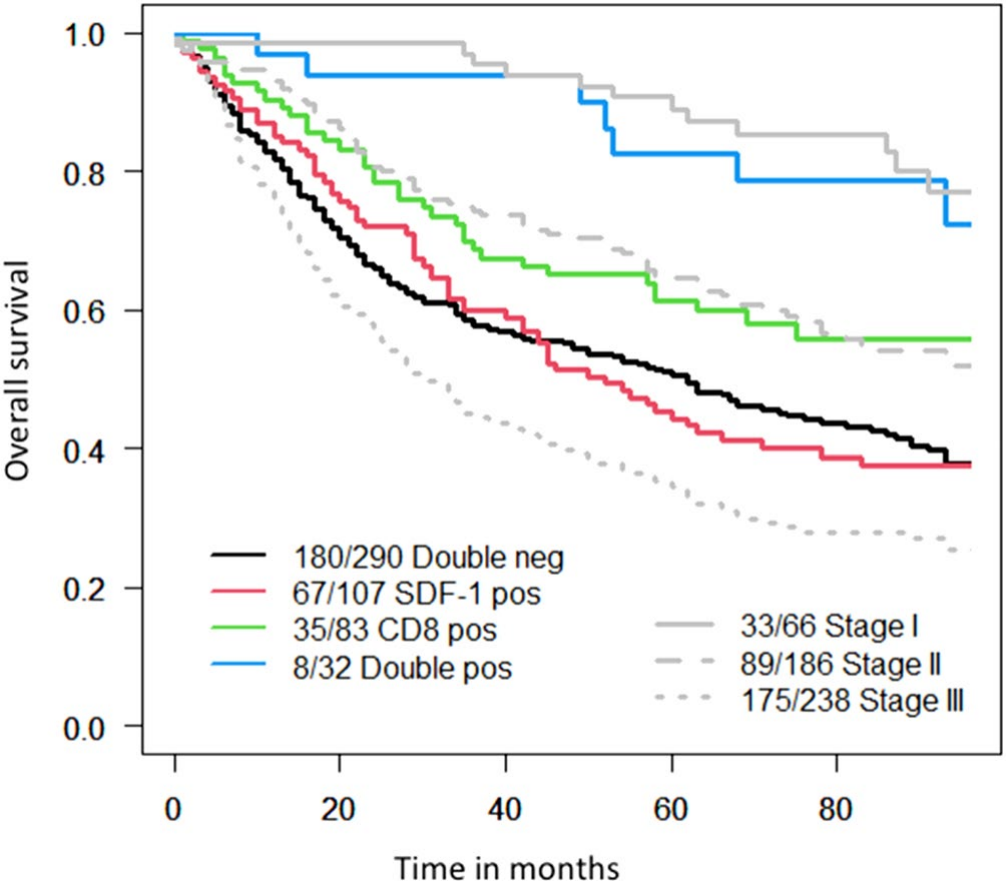
Kaplan–Meier survival curves of the overall cohort with the pairwise comparisons between the different curves. Effects of combined high CD8+ T-cell density and SDF-1 tumor expression on overall survival in patients with CRC. Kaplan–Meier overall survival curves were designed according to SDF-1 tumor expression and CD8+ cell density in patients bearing CRC as indicated. Cut-off values established by regression tree analysis were 200 for SDF-1 and 10 cells/punch for CD8+ cell infiltration^30–32^. Cumulative effects of SDF-1 expression and CD8+ cell density were explored. Black line indicates tumors with low CD8+ T-cell density and low SDF-1 expression. Red line refers to tumors with low CD8+ T-cell density and high SDF-1 expression. Green line indicates tumors with high CD8+ T-cell density and low SDF-1 expression. Blue line refers to tumors with high CD8+ T-cell density and high SDF-1 expression. In each curve is also represented in the background the overall survival according to the stage (light grey). Stage I is represented with a solid line, stage II with a dashed line and stage III with a dotted line. The pairwise comparisons between the different curves showed the following p-values: SDF-1^negative^/CD8^negative^(black) versus SDF-1^negative^/CD8^positiv^(green) *p* = 0.015. SDF-1^negative^/CD8^negative^(black) versus SDF-1^positive^/CD8^negative^(red) *p* = 0.89. SDF-1^negative^/CD8^negative^(black) versus SDF-1^positive^/CD8^positiv^(blue) *p* = 0.001. SDF-1^positive^/CD8^negative^(red) versus SDF-1^negative^/CD8^positiv^(green) *p* = 0.020. SDF-1^positive^/CD8^negative^(red) versus SDF-1^positive^/CD8^positiv^(blue) *p* = 0.001. SDF-1^negative^/CD8^positive^ (green) versus SDF-1^positive^/CD8^positiv^(blue) *p* = 0.065.

The 5-years survival rates were significantly different depending on the nature of immune infiltration (Table 2). Most importantly, Kaplan–Meier plots clearly indicated that 5-year survival rate was significantly better in cases of CRC with high expression on SDF-1 and CD8+ T-cell infiltration compared to tumors showing a high infiltration of CD8+ T-cells only 66% (95% CI 48–79%) versus 55% (95% CI 45–64%); *p* = 0.0004. In order to clarify the differences between all four groups, we calculated pairwise for each two curves the log rank *p* values and also took into account the multiple testing according to the Benjamini and Hochberg method. After performing a separate analysis we came to the following results Fig. 2.

**Figure 3:**
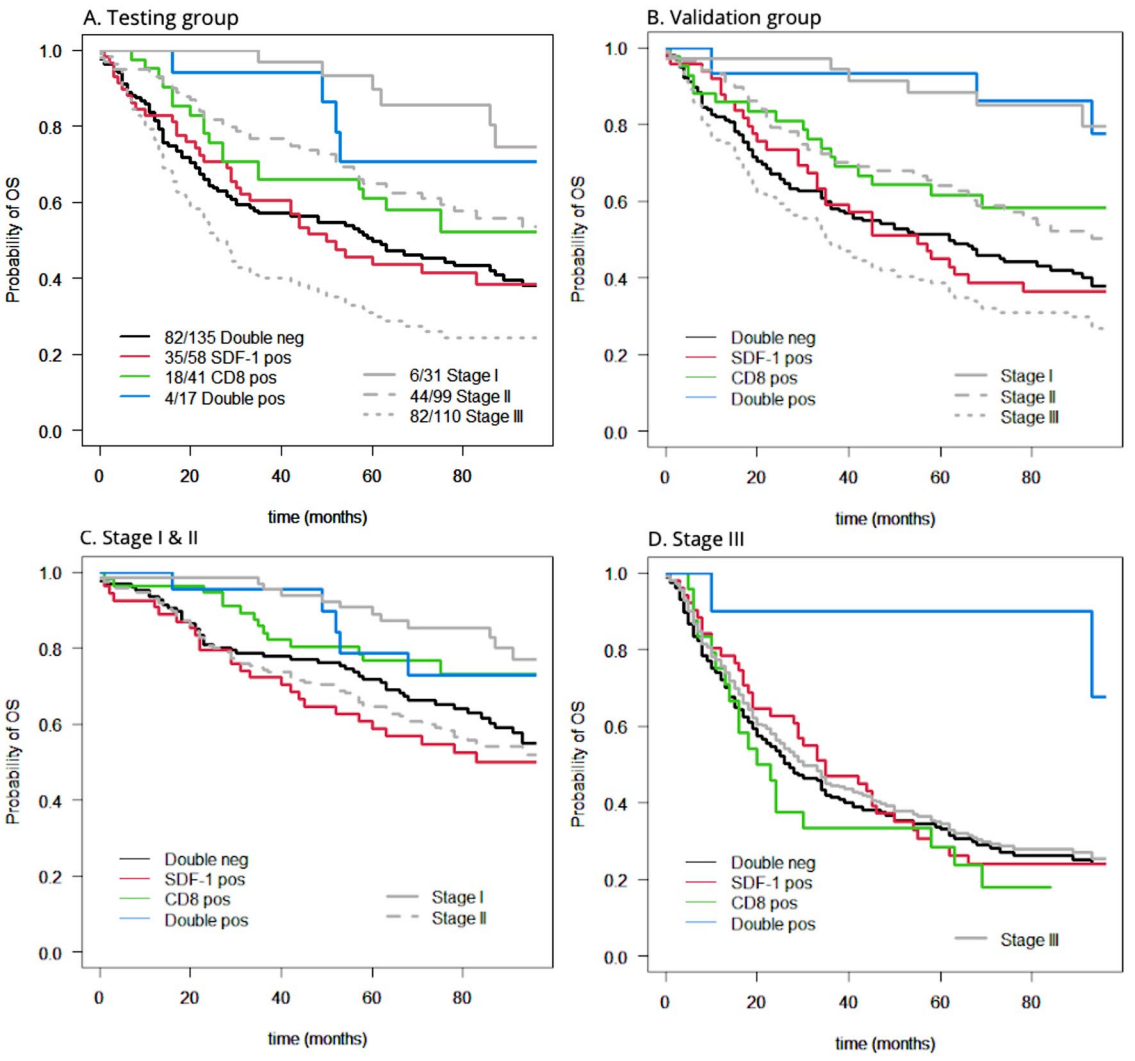
Kaplan–Meier overall survival curves according to CD8+ T-cell density and SDF-1 expression after stratifying the patients in testing and validation groups as well as in nodal negative and nodal positive groups. Kaplan–Meier overall survival curves after stratifying the patients in testing and validation groups as well as in nodal negative and nodal positive groups. (**A**) (Testing group): in this group the overall *p* value is 0.040 and the survival of patients with double positive markers is the only one, which (almost) reaches *p* values < 0.05 in comparison to the case with negative CD8+ T-cells. (**B**) (Validation group): Here the overall *p* value is 0.009. The survival of patients with double positive markers is the only one that reaches *p* values < 0.05 in comparison to the case with negative CD8+ T-cells. (**C**) (Stage I&II): No combination reaches the adjusted for multiple comparison *p* values < 0.05, even though overall *p* is 0.04. (**D**) (Stage III): With an overall *p* value of 0.02, the combination of high density of CD8+ T-cells and high expression of SDF-1 has an outstanding positive impact in the survival of patients with CRC at stage III.

Furthermore, we divided randomly our cohort in two groups, a testing group and validation group that also showed this difference between the groups (Fig. 3A, B). Since the patients with positive lymph nodes are associated with a worse prognosis, we also stratified the patients according to the nodal status (Fig. 3C, D). Interestingly, we found that the synergistic prognostic effect of CD8+ T-cells and SDF-1 was mainly present in the nodal positive group (Stage III) with a *p* value of 0.009.

### Univariate and multivariate analysis of SDF-1 expression and CD8+ T-cell infiltration by tumor cells and tumor-infiltrating immune cells

Univariate Cox regression analysis revealed that the combination of high expression of SDF-1 and high CD8 + T cell infiltration is significantly associated with an increased overall survival (HR = 0.34; 95% CI 0.17–0.66; *p* = 0.002). Age, male gender, tumor grade, T-stage, N-stage, invasive margin and vascular invasion were all significantly associated with a poor prognosis in univariate analyses (Table 4).

**Table 4:** Uni- and multivariate Hazard Cox regression survival analysis considering the combination of both markers (n = 613 and n = 576, respectively).

	Univariate				Multivariate			
	HR	95%CI		*p* value	HR	95%CI		*p* value
Age	1.03	1.02	1.04	**< 0.001**	1.04	1.03	1.05	**< 0.001**
Gender (male vs female)	1.53	1.23	1.90	**< 0.001**	1.61	1.28	2.02	**< 0.001**
pT (high vs low)	3.42	2.32	5.04	**< 0.001**	2.41	1.57	3.71	**< 0.001**
pN (high vs low)	3.28	2.61	4.14	**< 0.001**	2.36	1.84	3.02	**< 0.001**
Grade (high vs low)	5.31	1.32	21.33	**0.019**	2.88	0.69	11.95	0.146
Vascular invasion	2.49	1.99	3.12	**< 0.001**	1.99	1.56	2.53	**< 0.001**
Invasive margin	1.92	1.48	2.50	**< 0.001**	1.41	1.06	1.88	**0.017**
MMR status	1.53	1.07	2.19	**0.021**	1.32	0.91	1.92	0.149
SDF-1^high^/CD8 + ^low^	1.09	0.84	1.42	0.526	1.18	0.89	1.55	0.247
SDF-1^low^/CD8 + ^high^	0.66	0.47	0.92	**0.015**	0.89	0.62	1.29	0.549
SDF-1^high^/CD8 + ^high^	0.34	0.17	0.66	**0.002**	0.45	0.23	0.89	**0.021**

Multivariate analyses showing Hazard Ratios and *p* value for all CRCs (n = 576 less than 613 due to missing values) conferred by SDF-1 expression and CD8 + cell density, age, sex, tumor size, lymph node involvement, tumor grade, vascular invasion, tumor border configuration and microsatellite stability^33^. Significant *p* values are bold.

In a multivariate Hazard Cox regression survival analysis the combined high expression of SDF-1 and high CD8 + T-cell infiltration in CRC succeeded to retain its role as an independent prognostic factor for overall survival (HR = 0.45, 95% CI 0.23–0.89; *p* = 0.021). Moreover, we found that an increased age (HR = 1.04; 95% CI 1.03–1.05; *p* < 0.001), male gender (HR = 1.61; 95% CI 1.28–2.02; *p* < 0.001), a higher T-stage (HR = 2.41; 95% CI 1.57–3.71; *p* = 0.001), N-stage (HR = 2.36; 95% CI 1.84–3.02; *p* < 0.001), vascular invasion (HR = 1.99; 95% CI 1.56–2.53; *p* < 0.001) and invasive margin (HR = 1.41; 95% CI 1.06–1.88; *p* = 0.017) were independently associated with a poor prognosis (Table 4).

## Discussion

A significant amount of studies showed that the infiltration of CRC by CD8+ T-cells represents a favorable prognostic factor for the clinical outcome. Since then, more and more scientists are exploring the role of the microenvironment and immune response in the development of malignancies^11–20^. A great variety of immunocompetent cells, cytokines, and chemokines are currently investigated. Our team has already tested some of these factors in previous studies^34–36^. Furthermore, a study of Pagès et al.^44^ from May 2018 showed that the Immunoscore could determine the risk of recurrence in patients with colon cancer.

Among other immune markers, several studies have investigated the expression of SDF-1 and its role in tumor immunobiology. However, these studies came to conflicting data. Some of them showed that the high expression of SDF-1 is associated with reduced overall survival in patients with lung, pancreatic, and esophagus-gastric cancer. In contrast to these results, it was observed that in breast cancer, the high expression of SDF-1 was associated with increased overall survival^37–40^. In the case of colorectal cancer, there is a high heterogeneity across existing studies^26–29^.

Some of the cells that produce SDF-1 are the endothelial and bone marrow cells, mucosal epithelial cells, tumor cells, and T-lymphocytes^41^. SDF-1 expression is increased in tissues characterized by neo-angiogenesis and inflammation, supporting chemotactic gradients attracting immune cells. In a previous study of our team, we investigated the SDF-1-CXCR4 chemokine axis in cell trafficking as well as in tumor progression^42^. In that study, we showed that the activation of CXCR4, which is suggested by the presence of its phosphorylated form (pCXCR4), in CRC tumors and in infiltrating immune cells is associated with a significant favorable prognosis. According to our data, Stanisavljevic et al.^29^ have also shown that SDF-1 expression represents a favorable prognostic factor for disease-free survival in CRC.

In the presented study we explored these two markers in CRC and investigated the role of each of them alone and in combination. We found that a better prognosis characterizes CRC showing high CD8+ T-cells density with high SDF-1 tumor expression in contrast to CRC having high CD8+ T-cell density only, most likely due to an effective antigen-specific immune response. Knowing that patients with metastatic disease in the lymph nodes have a worse prognosis compared to patients with negative nodes, we stratified our cohort in nodal positive and nodal negative groups and found that the combination of the two markers had mainly a prognostic impact in the nodal positive group. Since this group has a much worse prognosis compared to Stages I and II cancers, it is imperative to find better methods of evaluation and subsequently treatment for this group. Therefore this finding may help in this direction, since it seems that the combination of high CD8+ T-cells and high SDF-1 expression is associated with a significantly better prognosis. Our data provide novel insights into the prognostic relevance of the interaction between the innate and adaptive immune system in CRC microenvironment. Therefore our results could be the reason to design studies that explore the combination of CD8+ T-cells density with high expression of SDF-1 in other types of cancer (for example lung, pancreatic and esophagus-gastric cancer), where current data already showed reduced overall survival in the single marker analysis of SDF-1. For instance, Roy et al. showed in an experimental model of pancreatic cancer that SDF-1 expression inhibited tumor growth and cancer cell metastasis formation through cell cycle arrest, resulting in increased overall survival, conflicting the existing data about SDF-1 expression and prognosis in pancreatic cancer^43^.

Finally, we were able to identify a panel of immune markers with modest to strong correlation on a gene expression level (r > 0.39): CD163, MMP2, CD4, CD11b, CD45, CCL21, CD56, CD11c, CD18, MMP9, CD16A, IL-10, CCL7, CCL19, CCL11, CXCR4, FOXP3, CCL8, CCL23, CD14, CCL18, TGF-beta, CCL13 and T-bet and we found a strong upregulation of MMP-2, HLA-DR and CD14 in the SDF-1^high^/CD8 + ^high^ group indicating possibly an effective antigen-specific immune response in this patient subgroup.

When it comes to limitations, we have to begin with the fact that our study is a retrospective one. Nevertheless, by using the data that emerge from extensive retrospective analyses, we may, in the future, be able to develop prospective studies. Secondary, TMA technology may fail to represent tumor tissue heterogeneity. Yet, the blocks included in our TMA were derived from tumor centers and included more than 50% of cancer cells. Additionally, the large number of individual CRC specimens (> 600) may partly compensate for the heterogeneity of the immune contexture in different tumor areas. Finally, the group investigated in this study includes CRC patients that were operated between 1985 and 1998. At that time, the use of neoadjuvant therapy was not part of the treatment of CRC.

## Conclusions

Our data show for the first time that the combination of high CD8+ T-cell density with SDF-1 expression represents an independent, favorable, prognostic condition in nodal positive CRC, thereby shedding new light on the biological role of CD8+ T-cells and SDF-1 in colorectal cancer progression. With this side, we provide novel insights into the prognostic role of the immune microenvironment in CRC and raise a number of points, which might have a significant impact on clinical decision-making. Our finding might help to pave new avenues towards the development of novel treatment modalities by modifying the tumor immune microenvironment in CRC patients, especially in the context of personalized medicine.

## Data Availability

The datasets used and/or analyzed during the current study are available from the corresponding author on reasonable request.
